# Combating Venereal Diseases

**Published:** 1923-03

**Authors:** 


### COMBATING VENEREAL DISEASES.

We have received a copy of the seventh annual report of the National Council for Combating Venereal Diseases (80 Avenue Chambers, Southampton Row, W.C.1). The figures of cases presenting themselves for the first time at clinics for the last four years show a steady increase for three years and a decrease for the last year.

### What the Decrease Means.

Discussing the meaning of this recent decrease, the report says:—

"We believe that we are justified in assuming that the decrease shown during the last year is a real decrease in the incidence of disease, in that the decrease is most apparent in those localities where facilities for treatment and extensive public enlightenment have been provided, but a surer indication would have been given had returns been available as to the stage of the disease in which patients first presented themselves for treatment. In Denmark it is claimed that concealment of venereal disease does not exist and a low incidence has been obtained. This claim is based on the fact that new cases coming for treatment are all in the early stages, and but few late stages of the disease are seen in new patients. No similar information is available for this country, therefore we cannot say with absolute certainty that the decrease is actually a decrease in the number of new cases of disease, although there is certainly ground for assuming that this is the case."

### How to Secure Continuous Treatment.

There is still great difficulty in securing continuous treatment for the patients, a large proportion of whom still discontinue treatment while in an infective condition. The Council has, during the last year, endeavoured to obtain information as to the results of the various systems of compulsory continuous treatment in foreign countries and in overseas Dominions. Opinion in this country is undoubtedly in favour of a voluntary system.

"The American system of notification by number and compulsory detention of infective persons, like various Continental systems, gives evidence of grave abuse in that there is a tendency in some places to administer such legislation in a manner resulting in a form of regulated prostitution, in that the compulsory clauses are exercised in the main in relation to women, and in relation to women employed in prostitution. In countries where this is the case the compulsory measures for the control of venereal disease have replaced legislation for the regulation of prostitution, and the administrative machinery used for the one has been transferred to the other. In Sweden, Finland and Denmark the Sanitary Police are responsible for the tracing of cases of venereal disease and bringing them for examination and treatment."

### Overseas Legislation.

On the other hand, legislation more in accordance with the principles acceptable in this country has been adopted in our Dominions overseas:—

"Details have reached us from Canada as to the operation of Health Regulations in Ontario, which lead many people to believe that the situation in this country might possibly be improved by the adoption of some such measures. Before any compulsory measures can be taken in this country, unless they are limited to certain localities, it is necessary for the facilities for treatment to be greatly improved. Some of the clinics now opened are most deficient in accommodation, and large areas in the country are still unprovided with any clinics or adequate facilities for treatment."

